# Ultrasound-guided scalp nerve block in anesthesia of children receiving cranial suture reconstruction

**DOI:** 10.1186/s12871-023-02223-9

**Published:** 2023-08-01

**Authors:** Tianxiao Zou, Shenghua Yu, Guili Ding, Rong Wei

**Affiliations:** 1 Department of Anesthesiology, Children’s Hospital of Shanghai, Shanghai, China; 2 Department of SICU, Children’s Hospital of Shanghai, Shanghai, China

**Keywords:** Ultrasound-guided scalp nerve block, Craniosynostosis, Pediatrics, Anesthesia

## Abstract

**Objective:**

Analgesia is very important for children with craniosynostosis who are undergoing cranial suture reconstruction. This study investigated the effectiveness and safety of an analgesic technique based on scalp nerve block combined with general anesthesia versus general anesthesia alone.

**Methods:**

This was a single-center, prospective, randomized, controlled study. A total of 60 children aged 6-24 months who underwent cranial suture reconstruction were randomly divided into two groups: Group A (general anesthesia combined with scalp nerve block) and Group N (general anesthesia). The hemodynamics were recorded preoperatively, at 5 min after incision and at 1, 6 and 12 h after surgery; the pain was scored at 1, 6 and 12 h after surgery, and blood glucose was detected at 1 h after surgery.

**Results:**

The mean arterial pressure and heart rate at 5 min after incision and 1 h after surgery in Group N were higher than those in Group A; the blood glucose and FLACC score in Group N were higher than those in Group A; and the number of postoperative analgesic pump presses were also significantly increased in Group N.

**Conclusion:**

Preoperative scalp nerve block can reduce hemodynamic fluctuation and postoperative pain in children undergoing cranial suture reconstruction for craniosynostosis. Thus, it can be safely and effectively applied in the anesthesia of these children.

## Background

Craniosynostosis is a relatively uncommon disease in pediatric neurosurgery that affects skull and brain development due to premature closure of the cranial suture [1]. Cranial suture reconstruction is the main treatment for craniosynostosis, but it is invasive, and therefore perioperative analgesia is particularly important. Effective analgesia can not only reduce hemodynamic fluctuation but also reduce postoperative pain and accelerate recovery [2]. This study investigated the effectiveness and safety of an analgesic technique based on scalp nerve block combined with general anesthesia versus general anesthesia in pediatric patients receiving cranial suture reconstruction for craniosynostosis [3]. Heart rate (HR), blood pressure (BP), and blood glucose were detected, and pain was scored to analyze the clinical effects of scalp nerve block in anesthesia for pediatric cranial surgery and postoperative analgesia.

## Methods

### Patients

The study was performed between Oct 1, 2021, and Oct 1, 2022. Written informed consent was obtained from the parents or relatives of patients. A total of 60 children (ASA I or II, aged 6 months to 24 months) who were scheduled for elective cranial suture reconstruction in the Department of Neurosurgery in Shanghai Children's Hospital were enrolled in this study. Exclusion criteria were as follows: hypersensitivity to ropivacaine, neurological disorders (except for craniosynostosis), local infection at the predesigned puncture site, diagnosis with other congenital disorders, participation in other clinical studies within 4 weeks before surgery, and abnormal electrocardiograph (ECG; presence of 1 or more of following features: bundle branch block, conduction delay, signs of right or left ventricular hypertrophy or left atrial enlargement, prolonged QTC interval, or ST-T depression exceeding 0.5 mm and/or pathological Q waves). Patients were randomly allocated into two groups by using computer-generated random numbers: Group A (general anesthesia combined with scalp nerve block) and Group N (general anesthesia). This was a single-center study designed according to the CONSORT statement and approved by the Institution Review Board (Ethics Commission of Children’s Hospital of Shanghai, Ref. No. 2021R029-F02; registered at the China Clinical Trial Registry Center, registration number ChiCTR2200066131).

### Anesthesia, surgery

After preoperative fasting (6 h for solid food and then 4 h for breast milk), anesthesia and monitoring were standardized for all children. ECG, pulse oximetry and end-tidal CO_2_ (ET CO_2_), and nasopharyngeal temperature were continuously monitored during anesthesia and recorded at a fixed interval of 5 min. General anesthesia was inducted with 0.01 mg/kg atropine, 0.1 mg/kg midazolam, 0.1 μg/kg sufentanil, 2 mg/kg propofol and 0.6 mg/kg rocuronium. An appropriate endotracheal tube with a visual laryngoscope was then inserted 2 min later. Pressure-controlled ventilation was employed. Anesthesia was maintained with 2% sevoflurane. Ultrasound-guided radial artery and femoral vein puncture was performed, and invasive arterial blood pressure and central venous pressure were monitored.

### Scalp nerve block

In Group A, an ultrasound-guided bilateral scalp nerve block was performed with 0.3% ropivacaine before incision. The total dose of local anesthetic was 3.6 mg/kg in each patient. The procedures for nerve block were as follows:

#### Supratrochlear nerve block

**Fig. 1:**
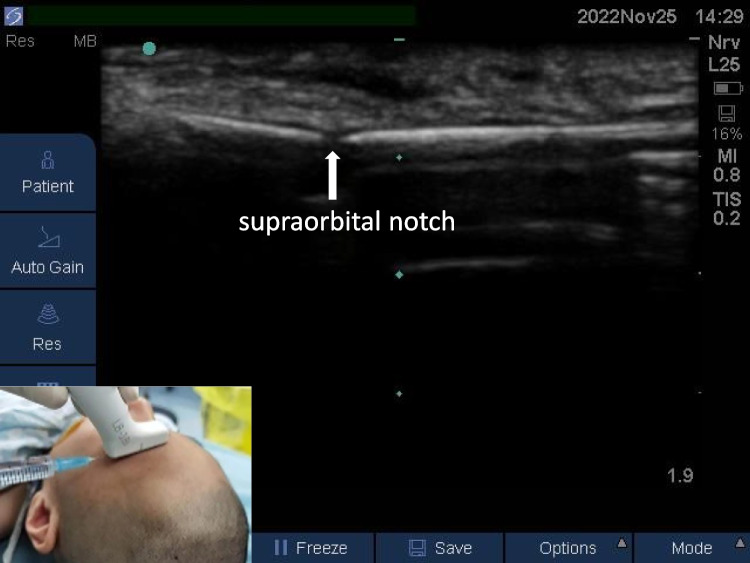
Supraorbital nerve and supratrochlear nerve block

The nerve block was guided with an S-NERVE ultrasound machine (Sono Site, USA), an ultrahigh frequency line array probe was placed parallel to the brow arch, the probe position was adjusted, and the supraorbital notch was found on the ultrasound image (an interruption of the bony surface echo). In-plane technology was used with an interior approach, 0.1 ml/kg local anesthetic was injected at the medial side of the supraorbital notch, and local anesthetic spread along the brow arch bone cortex (Fig. 1).

#### Supraorbital nerve block

When the supratrochlear nerve block was completed, the puncture needle continued parallel to the supraorbital notch. Then, 0.1 ml/kg local anesthetic was injected. Local anesthetic could be seen spread along the bone cortex of the brow arch and wrapped around the supraorbital notch (Fig. 1).

#### Auriculotemporal nerve block

**Fig. 2:**
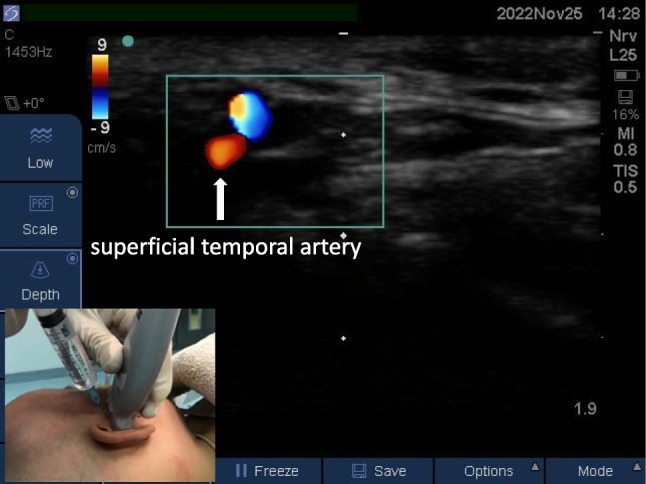
Auriculotemporal nerve block

The ultrasound probe was placed parallel to and above the zygomatic arch, and the superficial temporal artery was found 1 cm above the root of the zygomatic arch in front of the tragus. An ultrasound-guided out-of-plane technique was employed, and 0.1 ml/kg local anesthetic was injected into the deep surface of the superficial temporal artery (Fig. 2).

#### Lesser occipital nerve block

**Fig. 3:**
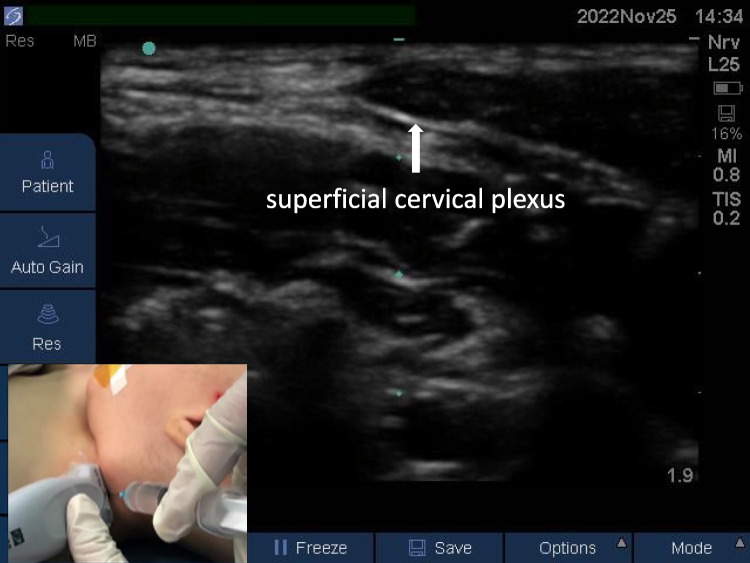
Lesser occipital nerve block

Due to the limitation of resolution of the ultrasound probe, the superficial cervical plexus was blocked to block the lesser occipital nerve. The child lay in a supine position with the head tilted to the opposite side. The superficial cervical plexus was positioned ipsilateral to the superficial cervical plexus and then punctured in-plane from the lateral side. The superficial cervical plexus (including the lesser occipital nerve) was located at the deep surface of the lateral border of the sternocleidomastoid muscle. Then, 0.1 ml/kg local anesthetic was injected, and the local anesthetic was observed to be spread in the interstitial space below the sternocleidomastoid muscle (Fig. 3).

#### Greater occipital nerve block

**Fig. 4:**
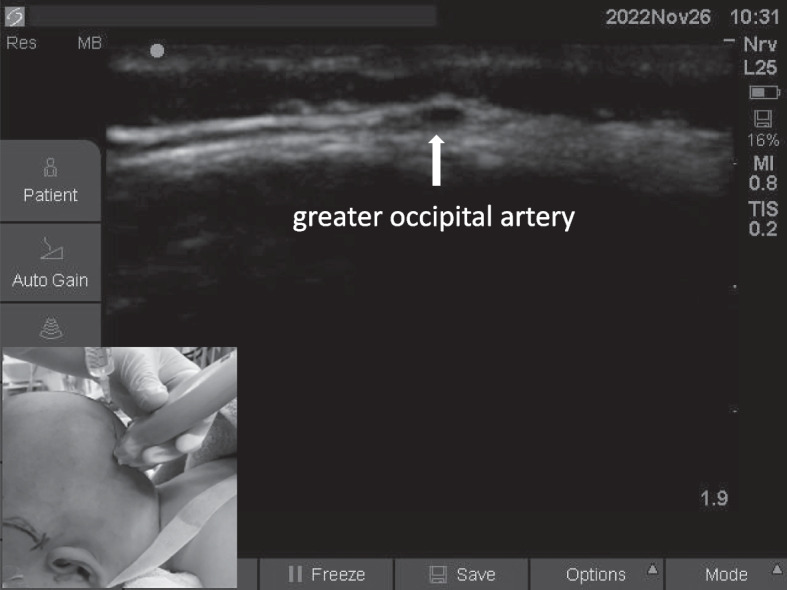
Greater occipital nerve block

The child was placed in a lateral position, and the ultrasound probe was placed parallel to the superior collar line. The greater occipital artery was found lateral to the external occipital ridge, and 0.2 ml/kg local anesthetic was injected into the medial artery with the in-plane technique (Fig. 4).

All anesthesia procedures were performed by the same experienced anesthetist, and the operations were completed by the same team of neurosurgeons. During the operation, if the mean arterial pressure (MAP) or HR rose by more than 30% of the baseline level, 0.1 μg/kg sufentanil was administered intravenously. The total amount of sufentanil during the operation was calculated.

### Postoperative pain relief

**Fig. 5:**
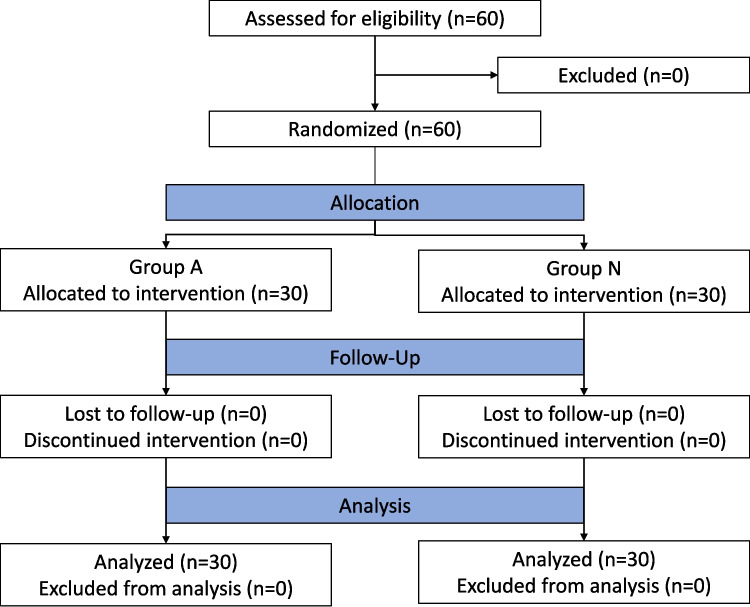
CONSORT flow diagram

After the operation, extubation was performed, and then the patients were transferred to the surgical intensive care unit (SICU). Patients in both groups received nurse-controlled analgesia (NCA) via an intravenous analgesia pump. The drug used for analgesia was 1 µg/kg sufentanil in saline (100 ml). The background dose was 0 ml, the NCA dose was 4 ml (sufentanil 0.04 μg/kg), and the lock time was 20 min. Pain was assessed by observing the face, legs, activity, cry, and consolability (FLACC). An FLACC score ≥ 4 indicated moderate pain, and the nurse pressed the analgesia pump to control the drug administration; an FLACC score ≥ 7 indicated severe pain, and oral paracetamol was administered for remedial analgesia. The consort diagram is shown in Fig. 5.

### Outcome measurements

The primary outcome objective of the study was the MAP, and the secondary objectives were the HR, FLACC scores, and venous blood glucose. The MAP and HR were recorded in 2 groups preoperatively (T_0_), 5 min after skin incision (T_1_), 1 h postoperatively (T_2_), 6 h postoperatively (T_3_) and 12 h postoperatively (T_4_). The FLACC score was obtained in 2 groups at T_2_, T_3_ and T_4_; venous blood glucose was detected at T_2_; intraoperative sufentanil consumption was calculated, and the total number of effective presses was recorded within 12 h postoperatively.

### Statistical analysis

Statistical analysis was performed with SPSS version 20.0 (SPSS, Inc., Chicago, Illinois, USA), and a value of *P* < 0.05 was considered statistically significant. Continuous variables are presented as the mean ± SD and were analyzed using one-way ANOVA with post hoc correction for multiple comparisons (Bonferroni correction) to determine differences among groups. Categorical variables were described as numbers (%) and were compared using chi-square tests. Biological data (venous blood glucose) and hemodynamic data (HR, MAP) were compared among groups and over time using repeated-measures ANOVA. Nonnormally distributed continuous variables, such as pain scores, are presented as the median and interquartile range (IQR, 25–75 percentile) and were analyzed with nonparametric tests (Kruskal–Wallis test and Mann‒Whitney U test with Bonferroni correction).

Sample size calculation was performed based on the assumption that a difference of 30% in MAP was clinically relevant. With an α of 0.05 and power of 80%, 26 patients in each group were needed. Therefore, we chose 30 patients per group in the study.

## Results

### Patient demographics and perioperative characteristics

Sixty children were included in this study. The results showed that there were no significant differences between the two groups in terms of sex, age, weight, height, duration of surgery and anesthesia, blood transfusion and loss (Table 1).

**Table 1:** General characteristics of children in the 2 groups (x¯±s)

Group	Gender (m/f)*/n*	Age (month)	Body weight (kg)	Height (cm)	Operation time (min)	Anesthesia time (min)
Group A	18/12	10.70±6.94	9.53±1.94	72.30±9.29	204.00±12.42	262.00±24.74
Group N	17/13	7.80±2.74	9.18±1.51	69.70±4.92	197.00±18.43	257.1±22.55
*P*	0.80	0.24	0.65	0.45	0.33	0.65

### Hemodynamic parameters (HRs and MAPs)

MAP and HR in the 2 groups at T_1_, T_2_, T_3_ and T_4_ were significantly higher than those at T_0_ (*P* < 0.05); MAP and HR in Group N at T_1_ and T_2_ were significantly higher than those in Group A (*P* < 0.05; Table 2).

**Table 2:** Perioperative hemodynamic parameters in the 2 groups (x¯±s)

Group	Time point	MAP (mmHg)	HR (bpm)
Group A	T_0_	70.20±2.62	120.10±5.60
	T_1_	78.00±1.63^*^	131.70±4.50^*^
	T_2_	75.50±2.46^*^	124.80±2.61^*^
	T_3_	77.30±0.95^*^	134.10±7.07^*^
	T_4_	77.60±1.95^*^	138.20±7.91^*^
Group N	T_0_	69.90±2.13	119.10±5.56
	T_1_	79.70±1.34^*#^	139.70±3.89^*#^
	T_2_	79.30±2.41^*#^	135.50±3.27^*#^
	T_3_	78.00±1.33^*^	130.40±4.45^*^
	T_4_	77.20±2.66^*^	139.00±7.73^*^

*MAP *mean arterial pressure, *HR* heart rate

*P<0.05 vs. T_0_. ^#^*P*<0.05 vs. Group A at the same time point

### Blood glucose, postoperative pain scores and analgesic consumption

The blood glucose in Group N was significantly higher than that in Group A; pain scores at T_3_ and T_4_ were significantly higher than those at T_2_ in the 2 groups. Moreover, compared to Group A, patients in Group N had significantly higher pain scores at T_2_ (Table 3).

**Table 3:** Postoperative blood glucose level and FLACC score in the 2 groups (x¯±s)

	Blood glucose (mmol/L)	FLACC pain score		
		T_2_	T_3_	T_4_
Group A	8.14±1.02	2.40±0.52	5.40±1.17	3.90±0.73
Group N	10.82±2.31	3.50±1.08	5.60±1.17	4.30±0.67
*P*	0.005	0.01	0.71	0.22

There was no significant difference in intraoperative sufentanil consumption between the 2 groups (P>0.05). The number of analgesic pump presses in Group N was significantly higher than that in Group A (*P* < 0.05) (Table 4).

**Table 4:** Intraoperative sufentanil consumption and number of times the analgesic pump was pressed in the 2 groups (x¯±s)

	Intraoperative sufentanil consumption (μg/kg)	Number of analgesic pump presses
Group A	0.46±0.05	2.70±0.94
Group N	0.48±0.06	4.40±1.87
*P*	0.49	0.02

### Pain control-related adverse events during the study period

The hemodynamics, oxygen saturation and respiratory rate were stable in both groups in the postanesthetic care unit (PACU). There were no complications, such as puncture site infection, local anesthetic toxicity, or hematoma, in any of the patients, and no remedial analgesia was administered for severe pain.

## Discussion

In cranial suture reconstruction procedures, skin incision and peeling of the periosteum are the most painful processes and may increase the patient’s blood pressure and HR [4]. General anesthesia is the most common type of anesthesia. Although intravenous sedatives and analgesics at higher doses can suppress intraoperative stress to certain extents, the adverse effects of opioids (such as nausea and vomiting, pupil contraction and other adverse reactions) may interfere with postoperative assessment of the pediatric nervous system and even lead to complications (such as respiratory depression) [5]. Scalp nerve block may suppress the conduction of peripheral pain signals to the central nervous system and reduce the amount of perioperative opioids, with no adverse effects (such as nausea or drowsiness) [6]. Since cranial suture reconstruction requires a long incision and may induce severe pain, ultrasound-guided scalp nerve block with general anesthesia is employed in children with craniosynostosis who are receiving cranial suture reconstruction as a new anesthetic method [7].

Craniotomy-related pain is mainly caused by skin incisions and muscle breaks rather than the operation on the brain parenchyma. The scalp is mainly innervated by the trigeminal nerve and the second and third cervical nerve roots [8]. The sensory nerves in the head that are more commonly chosen for clinical block include the supraorbital nerve, supratrochlear nerve, auriculotemporal nerve, greater occipital nerve and lesser occipital nerve. In this study, the HR and blood pressure of children in Group N were significantly higher than those in Group A at the beginning of surgery and 1 h postoperatively, indicating that ultrasound-guided scalp nerve block was effective in reducing surgical pain in Group A. Children in this study could not express pain as clearly as adults, and therefore, hemodynamics (e.g., MAP, HR) and blood glucose were measured, and the FLACC score was obtained to assess the effectiveness of scalp nerve block. Blood glucose is a relatively sensitive indicator of stress. When the body suffers trauma, the hypothalamic-pituitary-adrenocortical axis is excited and secretes a large amount of glucocorticoids, progressively increasing the plasma cortisol and blood glucose [9]. Our results showed that the blood glucose level in Group N was significantly higher than that in Group A at 1 h after the operation, indicating that scalp nerve block suppressed pain, reduced stress and inhibited the increase in blood glucose [10].

The MAP and HR at T_1_, T_2_, T_3_ and T_4_ were all higher than those before the operation in both groups. The postoperative FLACC score indicated that the children still had mild pain, indicating that scalp nerve block did not completely abolish pain during the cranial suture reconstruction. Cranial suture reconstruction differs from general craniotomy. The former is more extensive, and the incisions are made anteriorly to the supraorbital rim, posteriorly to the occipital region, and on both sides to the temporal region. The scalp is often crossed and coinnervated by multiple nerves [11]. In this study, ropivacaine was used to block the main nerves innervating the scalp in the surgical area, but some minor branches, such as the zygomaticotemporal nerve, were unblocked because blocking too many nerves would make the nerve block procedure too cumbersome. The FLACC scores at 1 h and 6 h after surgery in Group A were significantly lower than those in Group N, while the number of analgesic pump presses was markedly lower in Group A, suggesting that the analgesic effect of scalp nerve block extended to the postoperative period. Nerve block as the basis of multimodal analgesia, complemented by NSAIDs, is more effective in reducing postoperative pain, agitation and hemodynamic fluctuation and reduces the adverse effects of various drugs and analgesia [12]. Most studies related to scalp nerve block mainly focus on the intraoperative parameters, but our study indicated the prolonged analgesic effect of scalp nerve block after surgery, which is crucial for smooth postoperative recovery. A second scalp nerve block at the end of surgery can extend the duration of nerve block and provide better postoperative analgesia [12]. Therefore, the use of scalp nerve block in pediatric craniotomy can reduce intraoperative stress while providing some postoperative analgesia and helping speed up postoperative recovery [13, 14]. In addition, a good scalp nerve block can also be used for intraoperative awakening children receiving craniotomy [15]. Dexmedetomidine can be added to local anesthetic agents as an adjuvant in scalp block to prolong the duration of pain relief after craniotomy without bradycardia and hypotension [16].

However, there were still limitations in this study: the scalp sensory nerves are thin and difficult to distinguish on ultrasonography, and the identification of these nerves requires ultrasound-assisted localization of anatomical landmarks such as bone and blood vessels. However, the fact that there are variants of scalp nerves may affect the accuracy of nerve blocking. The amount of local anesthetic used may also affect the effectiveness of nerve block. In adult studies, the amount of local anesthetic used at each puncture site ranged from 2 to 5 ml. Few studies have been conducted to investigate the amount of local anesthetic in pediatrics, and the amount of local anesthetic is often reduced to avoid the risk of local anesthetic toxicity, which may also compromise the efficacy and duration of the nerve block. In the present study, the children undergoing cranial suture reconstruction were 6-24 months old, and their body weight was very low; thus, the dose of local anesthetic was calculated based on the body weight of each child. The scalp nerves were relatively superficial compared to other nerves, and less local anesthetic was needed. Therefore, the dose of local anesthetic was adjusted based on the body weight according to previously reported methods [4, 17].Due to the low body weight of the children, blood samples were collected at 1 h postoperatively to test the blood glucose levels and thereby reduce the number of blood samples. The children in this study were not stratified for further analysis due to the small sample size and narrow age span. The autonomic nervous system is not well developed in infants, and hence, using MAP to evaluate the anesthesia effect is not accurate enough. Indicators such as ANI, NOL and SPI are indeed validated measurements of pain. However, they were not measured due to the unavailability of instruments, which is also one of the limitations of this study.

## Conclusions

In summary, ultrasound-guided scalp nerve block based on anatomical localization is helpful to suppress surgery-related stress and the amount of local anesthetic used in children with craniosynostosis who are undergoing cranial suture reconstruction. Preoperative scalp nerve block may also attenuate hemodynamic fluctuation and is more effective for postoperative analgesia, which promotes postoperative recovery. More studies are needed to confirm our findings and investigate the clinical efficacy of ultrasound-guided scalp nerve block in children receiving other surgeries.

## Data Availability

The datasets are not publicly available but are available from the corresponding author upon reasonable request.
